# Comprehensive profiling of functional Epstein-Barr virus miRNA expression in human cell lines

**DOI:** 10.1186/s12864-016-2978-6

**Published:** 2016-08-17

**Authors:** Marjolein J. G. Hooykaas, Elisabeth Kruse, Emmanuel J. H. J. Wiertz, Robert Jan Lebbink

**Affiliations:** Department of Medical Microbiology, University Medical Center Utrecht, Utrecht, The Netherlands

**Keywords:** Epstein-Barr virus (EBV), Herpesvirus, microRNAs, miRNA reporter, miRNA sensor, Small RNA sequencing

## Abstract

**Background:**

Epstein-Barr virus (EBV) establishes lifelong infections in its human host. The virus is associated with a broad range of malignancies of lymphoid and epithelial origin, including Burkitt’s lymphoma, post-transplant lymphoproliferative disease, nasopharyngeal carcinoma and gastric carcinoma. During the latent phase of its life cycle, EBV expresses more than 40 mature miRNAs that are highly abundant in tumor cells and may contribute to oncogenesis. Although multiple studies have assessed the relative expression profiles of EBV miRNAs in tumor cells, data linking these expression levels to functional target knockdown are mostly lacking. Therefore we set out to systematically assess the EBV miRNA expression levels in EBV^+^ tumor cell lines, and correlate this to their functional silencing capacity in these cells.

**Results:**

We provide comprehensive EBV miRNA expression profiles of the EBV^+^ cell lines C666-1 (nasopharyngeal carcinoma), SNU-719 (gastric carcinoma), Jijoye (Burkitt’s lymphoma), and AKBM (Burkitt’s lymphoma) and of EBV^−^ cells ectopically expressing the BART miRNA cluster. By deep sequencing the small RNA population and conducting miRNA-reporter experiments to assay miRNA potency, we were able to compare the expression profiles of the EBV miRNAs with their functional silencing efficacy. We observe a strong correlation between miRNA expression levels and functional miRNA activity. There is large variation in expression levels between EBV miRNAs in a given cell line, whereas the relative expression profiles are well maintained between cell lines. Furthermore, we show that miRNA arm selection bias is less pronounced for gamma-herpesvirus miRNAs than for human miRNAs.

**Conclusion:**

We provide an in depth assessment of the expression levels and silencing activity of all EBV miRNAs in B- and epithelial cell lines of different latency stages. Our data show a good correlation between relative EBV miRNA expression levels and silencing capacity, and suggest preferential processing of particular EBV miRNAs irrespective of cell-type. In addition to encoding the largest number of precursor miRNAs of all human herpesviruses, EBV expresses many miRNAs precursors that yield two functional miRNA strands, rather than one guide strand and a non-functional passenger strand. This reduced strand bias may increase the size of the EBV miRNA targetome.

**Electronic supplementary material:**

The online version of this article (doi:10.1186/s12864-016-2978-6) contains supplementary material, which is available to authorized users.

## Background

MicroRNAs (miRNAs) are small RNA molecules of ~22 nucleotides in length that regulate gene expression by binding to target mRNAs in RNA-induced silencing complexes (RISC). Mechanisms for miRNA-mediated target gene downregulation include enhanced mRNA degradation, suppression of translation and in rare cases slicing of the mRNA at the target site (reviewed in [1]). In the last decade, many virus-encoded miRNAs have been identified, predominantly within the genomes of large dsDNA viruses such as herpesviruses. The two members of the gamma-herpesvirus subfamily that infect humans, Kaposi Sarcoma Associated Herpesvirus (KSHV, HHV-8) and Epstein-Barr virus (EBV, HHV-4), express large clusters of miRNAs during their latent phase (reviewed in [2]). Since very few viral proteins are expressed during latency, the expression of these miRNAs greatly expands the number of gene products available to manipulate the host cell. Indeed, EBV miRNAs play important roles in regulating EBV-induced B cell transformation [3–5] and downmodulate pro-apoptotic proteins such as PUMA [6], Bim [7], Bid [8], caspase 3 [5] and others [9]. Additionally, the EBV miRNAs contribute to immune evasion by targeting the NK cell ligand MICB [10], the chemokine CXCL11 [11] and the inflammasome component NLRP3 [12].

EBV establishes a lifelong infection in over 90 % of the adult human population, where it predominantly resides in a latent stage in the memory B cell compartment. During its life cycle, the virus passes through different phases that are characterized by distinct gene expression patterns [13]. After primary B cell infection, EBV gradually shuts down its protein expression. Initially, during the latency III stage, all EBNA (EBV nuclear antigens) and LMP (latent membrane protein) proteins are expressed. This is followed by the latency II stage where only EBNA1 and LMP proteins are expressed. During the subsequent latency I stage, protein expression is restricted to EBNA-1 alone. Through occasional reactivation and possibly lytic replication in epithelial cells of the oropharynx, EBV can spread to new hosts [14]. Although primary EBV infection usually proceeds asymptomatically, it is the causative agent for infectious mononucleosis. Furthermore, EBV infection is associated with a number of malignancies of epithelial, B cell and (occasionally) NK/T cell origin [13]. EBV-associated tumors display one of the three gene expression profiles described above. Burkitt’s lymphoma (BL) for instance, is characterized by a latency I program, although EBV frequently switches to latency III in vitro [15]. Hodgkin’s lymphoma (HL) and nasopharyngeal carcinoma (NPC) follow the latency II program, whereas gastric carcinoma (GC) is characterized by a latency I expression pattern, although occasionally LMP2A can be detected as well [16–18]. In EBV-associated lymphomas of immunosuppressed individuals, EBV expresses the largest set of latent genes (latency III) [19]. Also B lymphocytes transformed with EBV in vitro (lymphoblastoid cell lines, LCLs) display the latency III gene expression pattern [20].

Intriguingly, whereas viral protein expression is strictly regulated during the EBV latency stages, viral miRNAs are expressed throughout all stages of latency. EBV miRNAs are expressed in clusters and are named BART and BHRF1 miRNAs [21–23]. The BART miRNAs are located in introns of the latent BART transcripts (BARTs) that are expressed during all latency phases. Upon induction of the lytic cycle, the expression of the BARTs is increased, which coincides with a modest increase in mature BART miRNA expression levels [24]. The BHRF1 miRNAs lie upstream (miR-BHRF1-1) or downstream (miR-BHRF1-2, miR-BHRF1-3) the protein-coding BHRF1 gene and are mainly processed from latent EBNA transcripts that are highly expressed in latency III B cells. EBV reactivation results in expression of lytic BHRF1 mRNA from which miR-BHRF1-2 and miR-BHRF1-3 can be processed [24, 25]. The relative abundance of EBV miRNAs has been assessed in several human tissues and cell lines via deep sequencing, microarray, or qPCR techniques [8, 9, 24, 26–32]. Although insightful, these evaluations are limited to the relative expression profiles of EBV miRNAs without correlating this to their biological silencing potential. EBV miRNA targets have been identified by various approaches, including PAR-CLIP studies [9, 32], but a comprehensive analysis of the activities of all EBV miRNAs is lacking. Here, we set out to connect these two parameters experimentally. For this, we monitored miRNA activity of both strands from each EBV-encoded pre-miRNA in four different cell lines that cover the main latency stages of EBV. In parallel we performed small RNA deep sequencing in these same lines and correlated their relative expression level to functional target knockdown. Our data show a strong correlation between miRNA expression level and functional target knockdown. EBV miRNA expression profiles are highly consistent between cell lines, where miRNAs abundantly expressed in one cell line are also abundantly expressed in other EBV^+^ cell lines. Expression of the BART cluster in EBV-negative cells maintains the same hierarchy. Intriguingly, we noticed that there is considerably less strand bias in the expression of viral miRNAs as compared to human miRNAs: viral miRNAs have a higher incidence of expressing both miRNA strands in cells, thereby potentially enhancing the regulatory capacity of these miRNA genes.

## Results and discussion

### Functional expression profiling using miRNA reporters

**Fig. 1:**
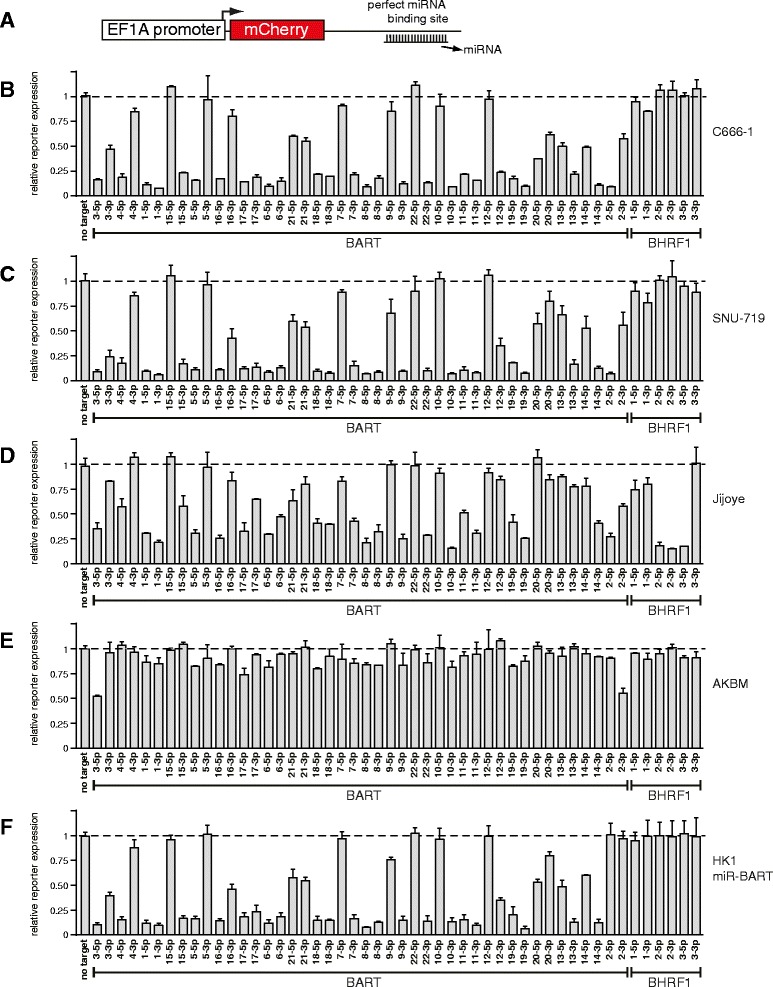
Functional EBV miRNA profiling using reporters. **a** Schematic representation of the lentiviral miRNA reporter construct used in this study **b**–**e** Relative miRNA reporter expression in **b** C666-1, **c** SNU-719, **d** Jijoye, **e** AKBM, and **f** HK1-miR-BART. Reporter expression was normalized to the empty control vector. miRNA binding decreases reporter expression; hence lower bars indicate higher functional miRNA expression. The displayed values are averages of two independent experiments performed in triplicate (+SD). The bars are shown in genomic order

In order to study the functional expression of EBV miRNAs in infected cells, lentiviral reporter constructs containing a single perfect miRNA binding site downstream of the fluorescent mCherry gene (Fig. 1a) were designed for all mature EBV miRNAs based on their sequences annotated in miRBase [33]. Although many miRNA hairpins are known to favor selection of one arm of the duplex over the other (the guide strand versus the star strand), both arms of a miRNA may be functional and capable of downregulating target mRNAs. We aimed at generating a comprehensive profile of the functional expression of all mature EBV miRNAs including the ones lacking miRBase annotation. To assess the functional expression of the latter ones, we designed miRNA reporters based on previous deep sequencing studies [34] or we introduced the complementary sequence of the entire miRNA arm including several adjacent nucleotides as a potential target site in our lentiviral mCherry reporter constructs. A complete list of the EBV miRNA sensor sequences is presented in Additional file 1.

We selected four EBV^+^ cell lines for functional screening: SNU-719 (type 1 EBV, gastric carcinoma, latency I/II, [35]), C666-1 (type 1 EBV, nasopharyngeal carcinoma, latency II), Jijoye (type 2 EBV, Burkitt’s lymphoma, latency III) and AKBM (type 1 EBV, Burkitt’s lymphoma, latency I, Akata strain). Additionally, in order to study EBV miRNA expression outside the context of virus infection, we analyzed EBV-negative HK1 (nasopharyngeal epithelial cell line) ectopically expressing the complete BART miRNA cluster but lacking BART2 (“HK1-miR-BART”). We next measured the functional downregulation of each miRNA reporter in these five cell lines (Fig. 1b–f). As expected, we observed differential downregulation of the miRNA reporter constructs, and these assessments were highly reproducible between experiments (Additional file 2). The majority of the BART miRNAs induced potent downregulation of reporters in the carcinoma cell lines (Fig. 1b–c). These findings are consistent with high expression levels of BART miRNAs in epithelial cells reported previously [8, 30, 36]. BART miRNA-mediated sensor downregulation in HK1-miR-BART cells was similar to that in EBV^+^ epithelial cells (Fig. 1f). For all miRNAs, at least one of the two strands induced potent target knockdown (>70 % repressed in the carcinoma cell lines), with the exception of miR-BART21 and miR-BART20 for which both strands induced only minor target downregulation (±50 %). In agreement with our data, large variations in expression levels between BART miRNAs have been observed previously [21, 29, 37, 38]; the mechanism behind this regulation remains to be determined.

The BHRF1 miRNAs are processed from EBNA-encoding transcripts initiated at the Cp or Wp promoter [21, 24], which are active during latency III. In addition, miR-BHRF1-2 and 3 can be processed from the 3’UTR of the BHRF1 mRNA, a lytic transcript that has also been detected in latent cells with an active Wp promoter [39]. Consistent with this, we barely see functional expression of BHRF1 miRNAs in carcinoma cell lines C666-1 and SNU-719, in which the Qp promoter drives expression of EBNA1 and the Cp and Wp promoter are not active (Fig. 1b–c). In contrast, in the latency III Jijoye cells, miR-BHRF1-2-5p, miR-BHRF1-2-3p and miR-BHRF1-3-5p potently downregulated their respective reporters (Fig. 1d). The miR-BHRF1-1-3p and miR-BHRF1-1-5p miRNAs do not show silencing activity in Jijoye cells. This is likely the result of a single nucleotide change in the 3p arm of the miR-BHRF1-1 pre-miRNA [40], which may preclude proper miR-BHRF1-1 expression.

The reporters for most of the miRNA strands absent from miRBase (miR-BART12-5p, miR-BART15-5p, miR-BART22-5p, miR-BHRF1-1-3p and miR-BHRF1-3-3p) were not or barely downregulated in the various cell lines tested, indicating that the registration of functional mature EBV miRNAs in this database is quite complete. However, although the miR-BART16-3p reporter was clearly downregulated in most cell lines, this miRNA is not annotated in miRBase and has not been analyzed in any of the previously published qPCR profiling studies of EBV^+^ samples.

**Fig. 2:**
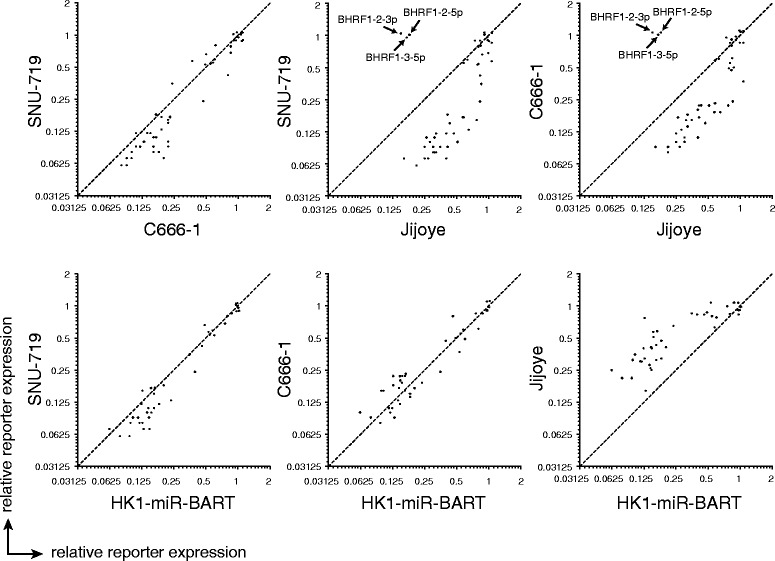
Comparison of functional miRNA expression between different cell lines. miRNA reporter expression shown in Fig. 1 was compared between the four EBV^+^ cell lines and the EBV^−^ cell line HK1-miR-BART (expressing the whole BART cluster except BART2). Every dot represents the relative reporter expression of a specific miRNA normalized to the control reporter. Since the BHRF1 miRNAs and BART2-3p and 5p were not included in the lentiviral vector introduced in the HK1 cell line, these miRNAs were not expressed in this cell line and are therefore not depicted in the HK1 plots. In AKBM the miRNA reporters were barely downregulated, making a comparison with the other cell lines less informative. These plots are shown in Additional file 3. The dashed line indicates the diagonal (no difference in relative reporter expression between both cell lines)

A strong correlation in BART miRNA reporter downregulation was observed between SNU-719, C666-1 and Jijoye (Fig. 2), i.e. in all three cell lines the same hierarchy of miRNA potency was observed between individual mature miRNAs. Remarkably, this relative expression profile was also maintained in the EBV-negative HK1 cell line expressing the BART miRNAs. This indicates that the main determinants for BART pre-miRNA processing, miRNA stability, RISC incorporation and target binding lie in the primary miRNA sequence (pri-miRNA) and are not affected significantly by viral sequences outside of the miRNA cluster or by cell-type specific expression of host cell genes. These findings are consistent with a study by Qiu et al., [31] in which EBV miRNA expression patterns were highly similar in tumor biopsies from various origins. Although we observed comparable miRNA expression patterns between cell lines, overall the BART miRNA reporters were less potently downregulated in the Jijoye cells as compared to the epithelial cell lines. This observation corresponds with a study by Cai et al. [21], where BART transcript levels were reduced in Jijoye cells as compared to C666-1 cells.

AKBM cells displayed a much-reduced potency of EBV miRNA activity as compared to the other cell lines; the majority of mature miRNAs induced only minor sensor downregulation (ranging from 0 to 25 %), whereas only miR-BART2-3p and miR-BART3-5p reduced mCherry expression by ±50 % (Fig. 1e, Additional file 3). Since these reporters acted distinctively as compared to the majority of EBV miRNA sensors, we tested whether they could be targeted by human instead of viral miRNAs. Although miR-BART2-3p and miR-BART3-5p reporters were not downregulated in EBV-negative HK1 cells (data not shown), we did observe a marked downregulation of the BART3-5p sensor in EBV-negative subclones of the Akata line which have lost the EBV genome (Additional file 4). This suggests that the observed regulation of this reporter in EBV-positive AKBM cells is likely caused by human miRNAs expressed in these cells, and not by viral miRNAs. The miR-BART2-3p reporter, however, was not downregulated in EBV-negative Akata cells, indicating that this miRNA may be functional in AKBM cells. It is unclear why miR-BART2-3p is more potent in AKBM cells as compared to other EBV miRNAs expressed in these cells.

### Expression profiling using deep sequencing

**Fig. 3:**
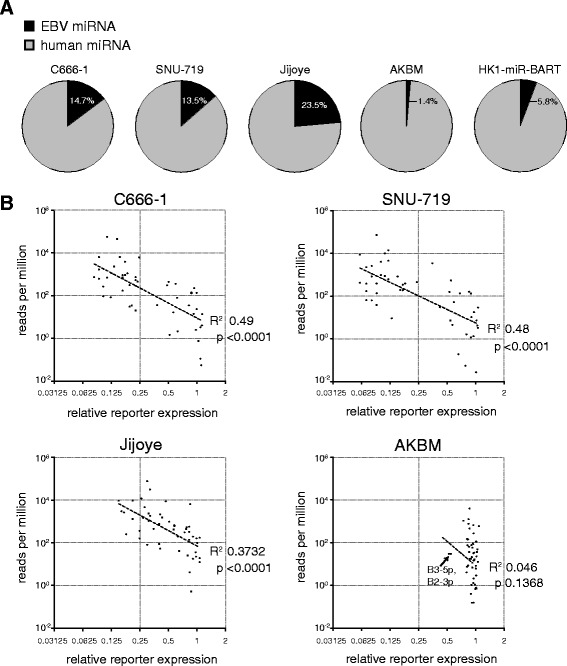
Correlation between EBV miRNA deep sequencing read counts and miRNA reporter expression. **a** Pie charts indicating the proportion of EBV and human derived miRNA sequences that mapped to miRNA loci for each indicated cell line. **b** Comparison of sequencing read counts (read count per million reads) to relative miRNA reporter expression (normalized to control reporter) in C666-1, SNU-719, Jijoye and AKBM. Each dot represents a single EBV miRNA. The dashed lines are linear regression curves of log-transformed read counts and reporter expression values

To allow correlation of our functional EBV miRNA profiling to mature miRNA expression levels, we next assessed the relative abundance of all EBV miRNAs by deep sequencing. This yielded 32–41 million sequencing reads per sample, after filtering of adapter sequences and low-quality reads (Table 1, Additional file 5). Reads were aligned to the human and EBV genome. The average read length was ~22 nucleotides, of which the majority mapped to miRNA sequences (75–97 %). The proportion of EBV reads ranged from 23.5 % in Jijoye to only 1.4 % in AKBM (Fig. 3a), suggesting that the poor miRNA reporter downregulation in the Akata-derived AKBM cells reflects low expression levels of the given miRNAs. To ensure that the relatively low expression of EBV miRNAs was not caused by loss of EBV from a large percentage of cells, we constructed a reporter vector in which 5 EBV miRNAs target sites were cloned downstream the mCherry reporter gene. Upon introduction of this ‘super-sensor’ in AKBM cells, we observed silencing in the majority of the cells which was not seen in EBV-negative subclones of the Akata cell line 2A8 and AK31 (Additional file 6), showing that relatively low EBV miRNA expression was not caused by absence of EBV from the AKBM cells.

**Table 1:** Summary of next generation sequencing results

	No. of reads		No. of miRNA reads		
cell line	total	genome mapped	human	EBV	% EBV reads
SNU-719	38,071,531	36,469,805 (95.8 %)	30,596,909	4,770,947	13.5 %
C666-1	38,248,224	36,080,697 (94.3 %)	28,912,402	4,972,225	14.7 %
Jijoye	40,677,253	34,107,346 (83.8 %)	20,372,508	6,260,036	23.5 %
AKBM	38,448,698	32,740,447 (85.2 %)	24,085,169	338,613	1.4 %
HK1-miR-BART	38,817,502	36,773,337 (94.7 %)	33,230,276	2,046,878	5.8 %

For the C666-1, SNU-719, and Jijoye cells, there was a clear inverse correlation between relative read counts (reads per million reads, RPM) and miRNA reporter expression (Fig. 3b). However, the correlation between these readouts within a single cell line (Fig. 3b) was less pronounced than the correlation of either read counts (Additional file 7) or reporter downregulation between two cell lines, e.g. the C666-1 and SNU-719 cells (Fig. 2). This difference may be explained by preferential deep sequencing of certain miRNA sequences; studies have indicated that miRNA sequencing bias can be introduced in the adapter ligation procedure, thereby favoring certain mature miRNA sequences over others [41]. Moreover, it is unlikely that the potency of individual miRNAs depends solely on the relative abundance of this miRNA. Indeed, the relative location of the miRNA target site in the 3’UTR impacts the potency of regulation [42]. In our experiments however, the distance between the mCherry reporter stop codon and the single miRNA target site is identical for all reporters. Besides target-site location, also seed-pairing stability (strength of the interaction) and target site abundance have been shown to influence miRNA activity [43].

We observed less miRNA reporter downregulation in Jijoye cells as compared to epithelial cell lines, although sequence read counts for given miRNAs were comparable (Figs. 1, and 3b, and Additional file 5). However, since we only assessed the relative EBV miRNA abundance (reads per million), it is possible that the Jijoye cells may actually have low absolute levels of EBV miRNAs. Indeed, previous studies report lower absolute BART miRNA expression levels in Jijoye as compared to C666-1 [21, 37]. Similarly, we observed roughly 2–3 fold lower relative EBV miRNA reads in the HK1-miR-BART cells as compared to the C666-1 and SNU-719 cells (Fig. 3a), although the potency of downregulation of the miRNA sensors was comparable between these three lines (Fig. 1). It is possible that the absolute EBV miRNA levels is similar between these lines.

### IsomiRs

Deep sequencing studies revealed that miRNAs can display extensive length and sequence variation [44]. These variants, termed isomiRs, are generated by differential Drosha/Dicer processing, trimming by exonucleases, non-templated nucleotide additions, and RNA editing events [45]. Indeed, we observed a large variety of isomiRs for all EBV miRNAs in each of the EBV^+^ cell lines analyzed. For many miRNAs less than 50 % of the sequenced reads had the exact canonical sequence as reported in miRBase (Additional file 5). Additionally, the most abundant sequence for several EBV miRNAs did not match the annotated sequence deposited in miRBase. The majority of isomiRs displayed 3’end variation, which is not expected to severely impact miRNA activity due to preservation of the 5’ end seed sequence. Nevertheless, the most abundant miR-BART10-3p sequence lacks a single 5’ nucleotide as compared to the canonical sequence in the EBV^+^ cell lines. Besides for miR-BART10-3p, high frequencies of isomiRs with alternative 5’ ends were also apparent for miR-BART2-3p, miR-BART16-3p and miR-BART5-5p (Additional file 5). Of these, only miR-BART10-3p and miR-BART5-5p represent the dominant arm of the miRNA duplex. For miR-BART5-5p, 38–60 % of the reads have a single nucleotide deletion at their 5’ end as compared to miRBase (Additional file 5). It is likely that these 5’ end variants will have an altered gene target repertoire as compared to the canonical miRNA sequences. Detection of the 5’ end variability prompted us to assess whether our miRNA reporters could be targeted by these isomiRs. Fortunately, only a minority of the reads had an elongated 5’ end that could obstruct activity towards our reporter construct. The relatively abundant 5’ variants of miR-BART10-3p, miR-BART2-3p, and miR-BART5-5p were truncated at their 5’ termini, rendering the reporters used in these studies still functional. For miR-BART16-3p, however, ~40 % of the sequences possessed a 2 nt extension at the 5’ end for which no complementary bases were present in our miRNA reporter construct. It is therefore possible that we underestimate the functional activity of this specific miRNA.

**Fig. 4:**
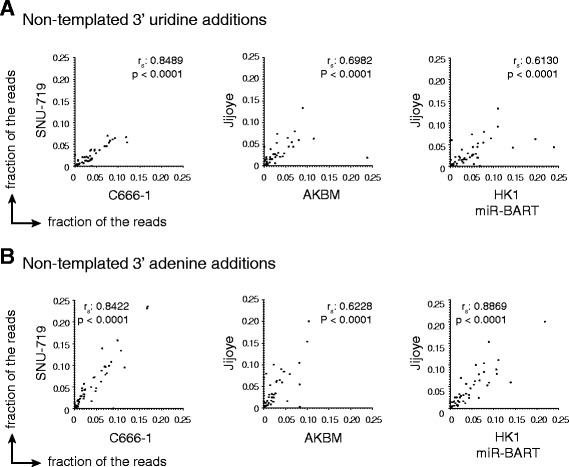
Frequencies of non-templated nucleotide additions to miRNAs correlate between cell lines. Comparison between the fractions of miRNA reads harboring non-templated adenine **a** or uridine **b** additions at their 3’ terminus in pairs of cell lines. Each dot represents a mature EBV miRNA. Presented are Spearman correlation coefficient r_s_ and p-value of correlations (two-tailed, Gaussian approximation)

Non-templated nucleotides can be added to precursor or mature miRNA 3’ termini by terminal nucleotidyl transferases (TNTs). The functional implications of these modifications are not fully understood, although miRNA uridylation and adenylation have been reported to impact miRNA stability [46–49] and potency [50, 51], and may act as a molecular signal to facilitate miRNA transport within and between cells [52]. We observed low levels of non-templated nucleotide additions (NTAs) to EBV miRNAs in our cell lines (ranging from 0 to 24 %) where adenine and uracil additions were most frequent. Intriguingly, we observed a clear correlation between the frequency of NTAs for given miRNAs between cell lines (Fig. 4a and b), where some EBV miRNAs were clearly preferentially adenylated or uridylated. In line with our findings, previous reports describe sequence specificity of terminal nucleotidyl transferases [48, 51, 53]. Whether these NTAs are important for EBV miRNA function remains to be determined.

### Reduced strand bias of gamma-herpesvirus miRNAs

**Fig. 5:**
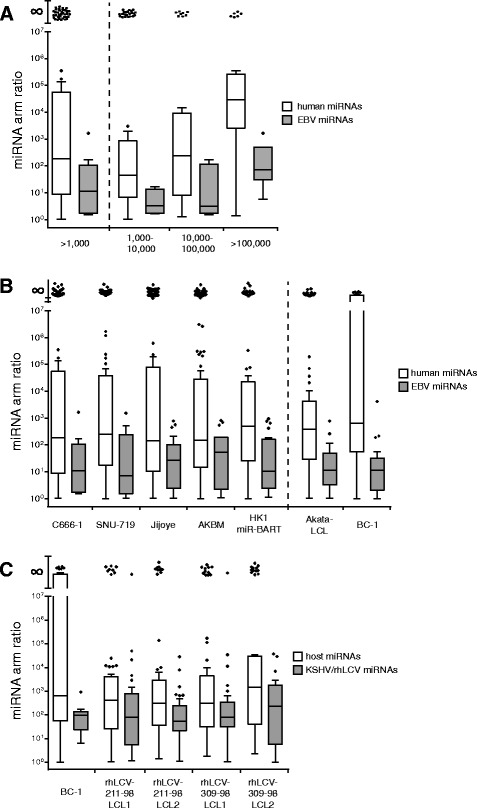
Gamma-herpesvirus miRNAs display less strand bias than host miRNAs. Box and whisker plots showing the miRNA arm ratios (deep sequencing read counts of the most abundant strand divided by the least abundant strand) in different cell lines. We limited the analysis to miRNAs with at least 1000 reads for the sum of both strands. The Tukey method was used for plotting the boxes, whiskers and outliers. miRNAs with one arm not detected are represented by a dot at the top of the graph (∞). **a** Arm ratios for human and EBV miRNAs in C666-1 cells. For the right six bars the miRNAs were divided in three different bins according to the read count number of the most abundant strand. **b** Arm ratios for human and EBV miRNAs in the EBV^+^ cell lines used in this study and EBV^+^ Akata-LCL and BC-1 cells (coinfected with EBV^+^ and KSHV^+^) deep sequenced in previous studies. **c** Arm ratios for KSHV/rhLCV and host miRNAs in BC-1 cells coinfected with EBV^+^ and KSHV^+^ and rhLCV infected rhesus macaque LCLs

Upon loading of a miRNA duplex onto Argonaute, one miRNA strand/arm (the guide RNA) is selected whereas the other one (passenger strand) is degraded [54]. For many miRNAs, there is a strong bias towards selection of one strand over the other, which results in profound differences in abundance of both miRNA strands within cells [55]. Although we saw a clear strand bias for human miRNAs, this phenomenon appeared much weaker for EBV miRNAs. For approximately half of the BART miRNAs, one of both arms was clearly dominant, whereas in a third of cases both strands potently downregulated their reporter (Fig. 1). This same feature was apparent in the deep-sequencing data: where for human miRNAs the median ratio between the most and least abundant miRNA strand was 1:186, for EBV miRNAs this was only 1:11 (Fig. 5a, left two bars). For a quarter of the EBV miRNAs there was a less than twofold difference between both strands, which applied to only 8 % of the human miRNAs. This difference in miRNA arm bias between human and EBV miRNAs was apparent for both high and low expressed miRNAs, as subdividing the miRNAs based on abundance yielded the same trend (Fig. 5a, right six bars). This phenomenon was not limited to C666-1 cells as we observed the same in SNU-719, Jijoye, and AKBM cells in our dataset and for Akata-LCL and BC-1 cells in sequencing data from other research groups (Fig. 5b).

We next extended our strand-bias analysis to non-EBV gamma-herpesvirus miRNAs (Fig. 5c). Intriguingly, also KSHV miRNAs expressed in BC-1 cells displayed reduced strand bias as compared to host miRNAs and the same was apparent for rhesus lymphocryptovirus (rhLCV, an EBV ortholog) miRNAs in rhesus monkey LCLs (Fig. 5b). Hence, it appears that gamma-herpesvirus miRNAs have retained or acquired the ability of both strands to functionally downregulate target genes.

The main feature determining arm selection is the thermodynamic stability of the miRNA duplex, whereby the miRNA arm with the least stable 5’ end is typically the guide strand that is loaded into RISC [56–58]. Also the secondary structure of the miRNA [59] and the identity of its first nucleotide [58, 60, 61] have been described to impact strand selection. How this reduced strand bias for gamma-herpesvirus miRNAs is obtained or retained and why this occurs remains to be determined. In principle, reducing strand bias may allow EBV to expand the number of target genes it regulates. Increasing the expression of miRNAs from one to two strands can be viewed as a genomic space-saving approach to enlarge regulatory capacity. This may be especially favorable for viruses since they are more constrained in their genome size than their host.

## Conclusions

We have provided comprehensive functional and relative expression profiles for all EBV miRNAs in four EBV^+^ cell lines representing different latency stages. In general, there is good concordance between the relative expression levels of miRNAs and their potential to silence target genes. The dominance of particular miRNAs over others is shared by all four cell lines and an EBV^−^ cell line in which the BART miRNAs were introduced through lentiviral transduction. This observation argues against an important role for cell-specific or virus-specific factors in regulating miRNA activity or abundance (apart from promoter usage), but rather suggests that the (pri-) miRNA sequence determines functional expression levels. Our deep sequencing datasets provide in depth information on EBV miRNA sequence diversity, including variation that affects miRNA target specificity by 5’end heterogeneity. Interestingly, EBV not only expresses the highest numbers of miRNAs of all human viruses; it also has increased regulatory capacity by functionally expressing both arms for a significant number of miRNAs.

## Methods

### DNA constructs

For the cloning of miRNA reporters, single perfect miRNA binding sites were introduced 29 bp downstream the mCherry reporter gene in the pSicoR-EF1a-PuroR-T2A-mCherry vector (described elsewhere, manuscript in preparation). See Additional file 1 for sequences cloned. Of note: the deep-sequencing analysis identified a single nucleotide change (position 17, T changed to C) in the mature miR-BART19-5p miRNA sequence in the C666-1 strain. Therefore, the miR-BART19-5p miRNA sensor used in the C666-1 cells to monitor miRNA activity does not fully match the miR-BART19-5p sequence expressed in these cells.

We ectopically expressed EBV miRNAs in EBV^−^ HK1 cells from the pLenti-Blast-eGFPintron vector (described elsewhere, manuscript in preparation). The miRNAs were cloned into an intron located in an eGFP marker gene driven by the EF1a promoter. Briefly, the EBV BART region was PCR amplified from genomic EBV DNA in three different PCR reactions (BART3 to 6, 21 to 18 and 7 to 14), which were subsequently combined into the pLenti-Blast-eGFPintron vector. All constructs were verified by Sanger sequencing.

### Cells and lentiviral transduction

Jijoye and 293 T human embryonic kidney cells were obtained from the ATCC (American Type Culture Collection). HK1 cells [62] were kindly provided by Prof. Dr. G. Tsao (The University of Hong Kong, Hong Kong). SNU-719 cells (originally derived from the Korean Cell Line Bank) and C666-1 were a kind gift from Prof. Dr. J. Middeldorp (VU University Medical Center, Amsterdam). As C666-1 cells can lose LMP1 and LMP2A expression in culture, the LMP1/2A expression status was verified by TaqMan qPCR which showed expression of both viral transcripts in our cells (data not shown). The EBV^+^ AKBM cell line [63] and EBV^−^ AK31 and 2A8 cell lines are derived from the Japanese Burkitt’s lymphoma cell line Akata. Cell lines were maintained in RPMI (Roswell Park Memorial Institute) 1640 supplemented with heat-inactivated 10 % fetal calf serum, 2 mM glutamine and antibiotics. Tissue culture flasks for culturing C666-1 cells were coated with fibronectin (Sigma F1141, [64]). Lentiviruses were produced via standard lentiviral production protocols using third-generation packaging vectors. Transductions of EBV^+^ cell lines were performed by spin infections at 1000 g for 1.5 h at 33 °C in the presence of 4 ug/ml polybrene and lentivirus. To obtain a pure population of BART miRNA expressing HK1 cells, lentivirally transduced cells were FACS sorted on a BD FACS Aria II based on eGFP expression.

### MiRNA reporter assay

Cells were lentivirally transduced at low MOI to achieve single lentiviral integrations, allowed to recover for ± 1 week, fixed, and analyzed by flow cytometry on a BD Canto II flow cytometer. FlowJo software (Treestar) was used for analyzing the flow cytometric data. The gating strategy consisted of selecting live cells (based on forward and side scatter) and then gating on mCherry-positive cells (thereby gating out untransduced cells). To calculate relative reporter expression, the mCherry geometric means of cells harbouring the miRNA reporters were normalized against those of cells harbouring the empty control vector.

### Construction of small RNA libraries and deep sequencing

RNA was isolated using Trizol according to manufacturer’s instructions (Life Technologies). Integrity of the RNA was verified using the Agilent 2100 Bioanalyzer (RIN scores were 10.0). Sequencing of small RNA was performed by BGI (Hong Kong, China), briefly: 3’ and 5’ adapters were ligated to size-selected RNA molecules (~18–30 nt length), followed by reverse transcription using SuperScript II (Invitrogen). cDNA was PCR amplified, after which clusters were generated using an Illumina cBot cluster amplification system. Single-end sequencing by synthesis was performed on the HiSeq 2000 platform using the TruSeq SBS Kit-HS V3. Good quality sequence reads were trimmed to remove adapter sequences and analyzed by using the sRNAbench 0.9 package [65]. Reads were aligned to the human (reference genome GRCh37) and EBV genome (NC_007605). Initially, no mismatches were allowed between the Illumina reads and the reference sequences. However, as we identified a single nucleotide change (position 17, T changed to C) in the mature BART19-5p miRNA sequence in the C666-1 EBV strain (as reported before by Chen et al. [34]), read count numbers for this miRNA were adjusted to reflect the correct miR-BART19-5p reads. We did not detect additional polymorphisms in any of the analyzed cell lines. Correlation coefficients were calculated using Graphpad Prism.

### MiRNA arm ratio calculations

miRNA arm ratios were calculated by dividing the number of reads of the most abundant arm by that of the least abundant arm, to obtain numbers between 1 (1 : 1 ratio) and infinite (if one arm was undetectable). Data from published studies were obtained from the NCBI Sequence Read Archive (http://www.ncbi.nlm.nih.gov/sra): Akata-LCL: SRR1003028 [66], BC-1: SRR343332 [67], rLCV-infected 211-98 rhesus macaque rLCL: run SRR1003031 and run SRR1003028, rLCV-infected 309-98 rhesus macaque rLCL: run SRR1003035 and run SRR1003033 [66]. Deep sequencing reads were analyzed by using the sRNAbench 0.9 package, single nucleotide mismatches to reference sequences were allowed in the analysis.

## Abbreviations

BL, Burkitt’s lymphoma; EBV, Epstein-Barr virus; GC, gastric carcinoma; HL, Hodgkin’s lymphoma; KSHV, Kaposi’s sarcoma associated herpesvirus; LCL, lymphoblastoid cell line; mRNA, messenger RNA; miRNA, microRNA; NPC, nasopharyngeal carcinoma; rhLCV, rhesus lymphocryptovirus; RISC, RNA induced silencing complex.

## Additional files

Additional file 1: List of miRNA sequences and perfect target sequences. List of miRNA sequences annotated in miRBase or (*) based on deep sequencing data from Chen et al. 2010 (column 2). Perfect target sequences (column 3) were cloned downstream of the mCherry reporter gene to monitor miRNA expression. ^#^ The deep-sequencing analysis in Fig. 3 identified a single nucleotide change (position 17, T changed to C) in the mature miR-BART19-5p miRNA sequence in the C666-1 strain. Therefore, the miR-BART19-5p miRNA sensor used in the C666-1 cells to monitor miRNA activity does not fully match the miR-BART19-5p sequence expressed in these cells (DOCX 20 kb) Additional file 2: Good reproducibility between miRNA reporter assays. Shown are the log-transformed relative reporter expression values of two independent experiments (each representing three technical replicates) in each of the four EBV^+^ cell lines. The data points are located close to the diagonal dashed lines, indicating good reproducibility (PDF 121 kb) Additional file 3: Comparison of functional EBV miRNA expression between AKBM and other EBV^+^ cell lines. miRNA reporter expression (data also shown in Fig. 1) was compared between AKBM and the three other EBV^+^ cell lines. Every dot represents the relative reporter expression of a specific miRNA as a percentage of the control reporter. The dashed line indicates the diagonal (no difference in relative reporter expression between both cell lines) (PDF 91 kb) Additional file 4: Assessment of miRNA reporters in EBV^−^ cells. Reporter activity for miR-BART2-3p, miR-BART3-5p and controls was compared between AKBM and the EBV^−^ Burkitt’s lymphoma cell lines, 2A8 and AK31 (AKBM data also shown in Fig. 1). The miR-BART2-3p reporter is only downregulated in AKBM but not in the other two cell lines, suggesting that downregulation of the reporter is specific for EBV^+^ cells and thus likely due to miR-BART2-3p expression. The miR-BART3-5p reporter was downregulated in all three cell lines, suggesting the influence of a common host cell factor such as a human miRNA on reporter expression (PDF 101 kb) Additional file 5: List of EBV miRNA sequence variants. The most highly abundant sequence, the normalized sequence read counts (reads per million genome-mapped reads), the percentage of canonical sequences and percentage of 5’ terminus variants of each EBV miRNA in the five cell lines are shown (XLSX 22 kb) Additional file 6: AKBM cells are EBV positive. A) Schematic representation of the EBV super-sensor. Five perfect EBV miRNA target sites were cloned downstream of the mCherry reporter gene. The vector allows for sensing of EBV presence by monitoring the combined functional expression of 5 EBV miRNAs. B) EBV-positive AKBM cells and EBV negative AK31 and 2A8 cells were either left untreated (cells alone), were transduced with an mCherry-control vector (control sensor), or were transduced with the EBV sensor from a). Cells were monitored for mCherry reporter expression at 7 days post transduction by flow cytometry (PDF 165 kb) Additional file 7: Comparison of deep sequencing reads between different cell lines. Deep sequencing reads were compared between the four EBV^+^ cell lines and the EBV^−^ cell line HK1-miR-BART. Every dot represents a single miRNA arm. BHRF1 miRNAs, BART2-3p and BART2-5p are not depicted in the HK1-miR-BART plots as these are not expressed in these cells. Pearson correlations R square and p-values are presented. Good correlations were observed between the read counts for BART miRNAs in all combinations of cell lines (PDF 157 kb)
