# Supplementary material for: Comprehensive profiling of functional Epstein-Barr virus miRNA expression in human cell lines
Source: BMC Genomics. 2016 Aug 17;17:644. doi: 10.1186/s12864-016-2978-6 (PMC4987988; doi:10.1186/s12864-016-2978-6)
Supplement: Additional file 1: — List of miRNA sequences and perfect target sequences. List of miRNA sequences annotated in miRBase or (*) based on deep sequencing data from Chen et al. 2010 (column 2). Perfect target sequences (column 3) were cloned downstream of the mCherry reporter gene to monitor miRNA expression. # The deep-sequencing analysis in Fig. 3 identified a single nucleotide change (position 17, T changed to C) in the mature miR-BART19-5p miRNA sequence in the C666-1 strain. Therefore, the miR-BART19-5p miRNA sensor used in the C666-1 cells to monitor miRNA activity does not fully match the miR-BART19-5p sequence expressed in these cells (DOCX 20 kb) [file 12864_2016_2978_MOESM1_ESM.docx]

**Additional file 1. List of miRNA sequences and perfect target sequences.**

| **miRNA** | **miRNA sequence (5’ to 3’)** | **target sequence (5’ to 3’)** |
| --- | --- | --- |
| BART1-3p | TAGCACCGCTATCCACTATGTC | GACATAGTGGATAGCGGTGCTA |
| BART1-5p | TCTTAGTGGAAGTGACGTGCTGTG | CACAGCACGTCACTTCCACTAAGA |
| BART2-3p | AAGGAGCGATTTGGAGAAAATAAA | TTTATTTTCTCCAAATCGCTCCTT |
| BART2-5p | TATTTTCTGCATTCGCCCTTGC | GCAAGGGCGAATGCAGAAAATA |
| BART3-3p | CGCACCACTAGTCACCAGGTGT | ACACCTGGTGACTAGTGGTGCG |
| BART3-5p | ACCTAGTGTTAGTGTTGTGCT | AGCACAACACTAACACTAGGT |
| BART4-3p | CACATCACGTAGGCACCAGGTGT | ACACCTGGTGCCTACGTGATGTG |
| BART4-5p | GACCTGATGCTGCTGGTGTGCT | AGCACACCAGCAGCATCAGGTC |
| BART5-3p | GTGGGCCGCTGTTCACCTAA (**)* | TTAGGTGAACAGCGGCCCAC |
| BART5-5p | CAAGGTGAATATAGCTGCCCATCG | CGATGGGCAGCTATATTCACCTTG |
| BART6-3p | CGGGGATCGGACTAGCCTTAGA | TCTAAGGCTAGTCCGATCCCCG |
| BART6-5p | TAAGGTTGGTCCAATCCATAGG | CCTATGGATTGGACCAACCTTA |
| BART7-3p | CATCATAGTCCAGTGTCCAGGG | CCCTGGACACTGGACTATGATG |
| BART7-5p | CCTGGACCTTGACTATGAAACA | TGTTTCATAGTCAAGGTCCAGG |
| BART8-3p | GTCACAATCTATGGGGTCGTAGA | TCTACGACCCCATAGATTGTGAC |
| BART8-5p | TACGGTTTCCTAGATTGTACAG | CTGTACAATCTAGGAAACCGTA |
| BART9-3p | TAACACTTCATGGGTCCCGTAGT | ACTACGGGACCCATGAAGTGTTA |
| BART9-5p | TACTGGACCCTGAATTGGAAAC | GTTTCCAATTCAGGGTCCAGTA |
| BART10-3p | TACATAACCATGGAGTTGGCTGT | ACAGCCAACTCCATGGTTATGTA |
| BART10-5p | GCCACCTCTTTGGTTCTGTACA | TGTACAGAACCAAAGAGGTGGC |
| BART11-3p | ACGCACACCAGGCTGACTGCC | GGCAGTCAGCCTGGTGTGCGT |
| BART11-5p | TCAGACAGTTTGGTGCGCTAGTTG | CAACTAGCGCACCAAACTGTCTGA |
| BART12-3p | TCCTGTGGTGTTTGGTGTGGTT (*) | AACCACACCAAACACCACAGGA |
| BART12-5p | ACCCGCCCATCACCACCGGACAG | CTGTCCGGTGGTGATGGGCGGGT |
| BART13-3p | TGTAACTTGCCAGGGACGGCTGA | TCAGCCGTCCCTGGCAAGTTACA |
| BART13-5p | AACCGGCTCGTGGCTCGTACAG | CTGTACGAGCCACGAGCCGGTT |
| BART14-3p | TAAATGCTGCAGTAGTAGGGAT | ATCCCTACTACTGCAGCATTTA |
| BART14-5p | TACCCTACGCTGCCGATTTACA | TGTAAATCGGCAGCGTAGGGTA |
| BART15-3p | GTCAGTGGTTTTGTTTCCTTGA | TCAAGGAAACAAAACCACTGAC |
| BART15-5p | AGGGAAACATGACCACCTGAAGTC (*) | GACTTCAGGTGGTCATGTTTCCCT |
| BART16-3p | ATCACCACCCTCTATCCATAT (*) | ATATGGATAGAGGGTGGTGAT |
| BART16-5p | TTAGATAGAGTGGGTGTGTGCTCT | AGAGCACACACCCACTCTATCTAA |
| BART17-3p | TGTATGCCTGGTGTCCCCTTAGT | ACTAAGGGGACACCAGGCATACA |
| BART17-5p | TAAGAGGACGCAGGCATACAAG | CTTGTATGCCTGCGTCCTCTTA |
| BART18-3p | TATCGGAAGTTTGGGCTTCGTC | GACGAAGCCCAAACTTCCGATA |
| BART18-5p | TCAAGTTCGCACTTCCTATACA | TGTATAGGAAGTGCGAACTTGA |
| BART19-3p | TTTTGTTTGCTTGGGAATGCT | AGCATTCCCAAGCAAACAAAA |
| BART19-5p | ACATTCCCCGCAAACATGACATG^#^ | CATGTCATGTTTGCGGGGAATGT^#^ |
| BART20-3p | CATGAAGGCACAGCCTGTTACC | GGTAACAGGCTGTGCCTTCATG |
| BART20-5p | TAGCAGGCATGTCTTCATTCC | GGAATGAAGACATGCCTGCTA |
| BART21-3p | CTAGTTGTGCCCACTGGTGTTT | AAACACCAGTGGGCACAACTAG |
| BART21-5p | TCACTAGTGAAGGCAACTAAC | GTTAGTTGCCTTCACTAGTGA |
| BART22-3p | TTACAAAGTCATGGTCTAGTAGT | ACTACTAGACCATGACTTTGTAA |
| BART22-5p | TGCTAGACCCTGGAGTTGAACC | GGTTCAACTCCAGGGTCTAGCA |
| BHRF1-1-3p | unknown | TTGTCAACCTCTTCAGGCCCGGGGTTAGTGATG |
| BHRF1-1-5p | TAACCTGATCAGCCCCGGAGTT | AACTCCGGGGCTGATCAGGTTA |
| BHRF1-2-3p | TATCTTTTGCGGCAGAAATTGA | TCAATTTCTGCCGCAAAAGATA |
| BHRF1-2-5p | AAATTCTGTTGCAGCAGATAGC | GCTATCTGCTGCAACAGAATTT |
| BHRF1-3-3p | unknown | ATTTTAACGAAGAGCGTGAAGCACCGCTTGC |
| BHRF1-3-5p | TAACGGGAAGTGTGTAAGCACA | TGTGCTTACACACTTCCCGTTA |
