# Supplementary figures and images for: Comprehensive profiling of functional Epstein-Barr virus miRNA expression in human cell lines
Source: BMC Genomics. 2016 Aug 17;17:644. doi: 10.1186/s12864-016-2978-6 (PMC4987988; doi:10.1186/s12864-016-2978-6)

C666-1

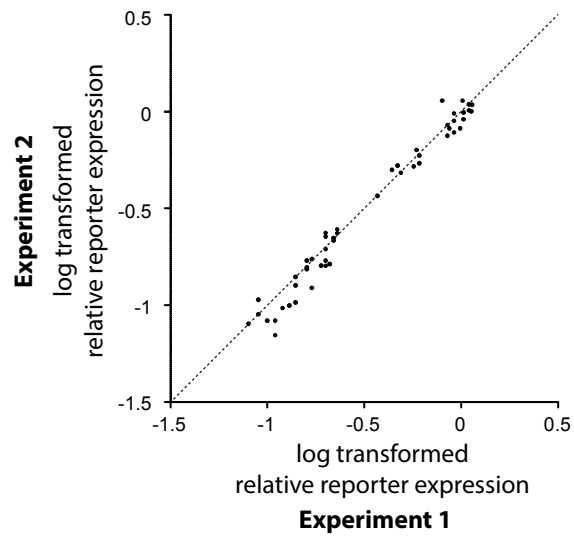

SNU-719

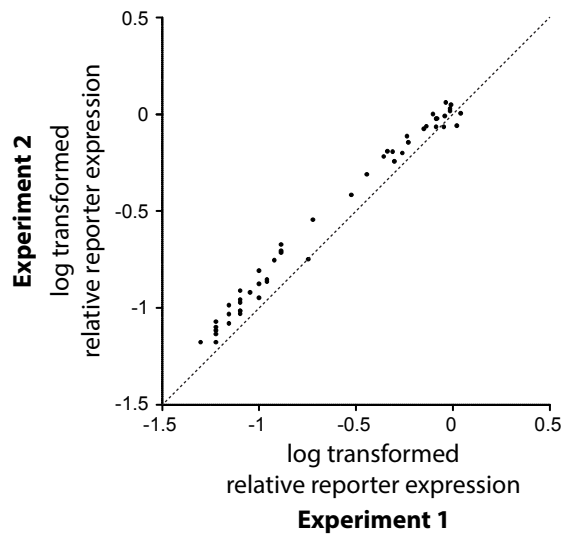

Jijoye

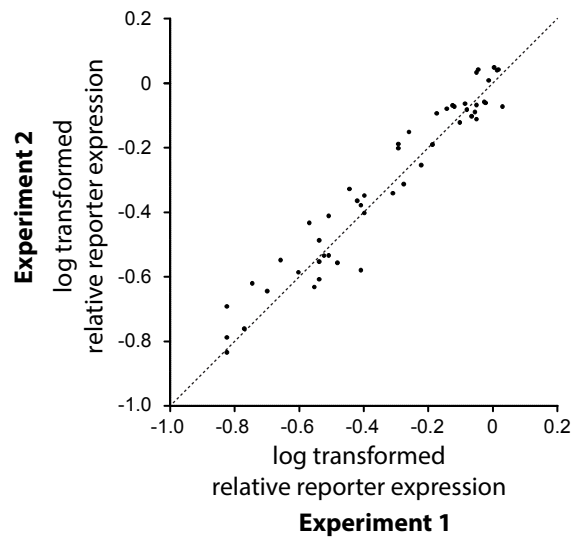

AKBM

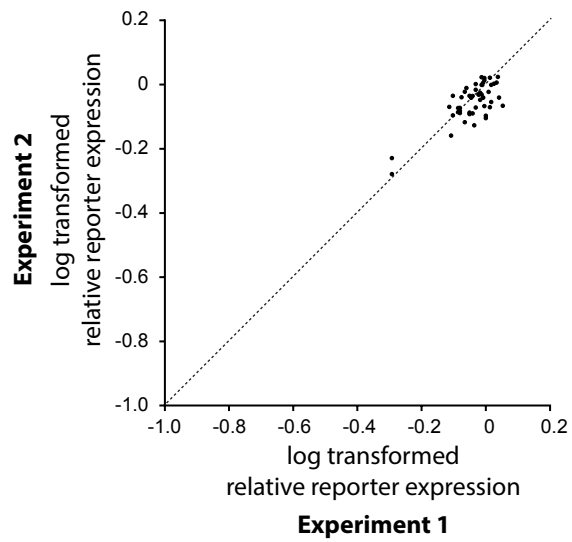

Supplement: Additional file 2: — Good reproducibility between miRNA reporter assays. Shown are the log-transformed relative reporter expression values of two independent experiments (each representing three technical replicates) in each of the four EBV+ cell lines. The data points are located close to the diagonal dashed lines, indicating good reproducibility (PDF 121 kb) [file 12864_2016_2978_MOESM2_ESM.pdf]

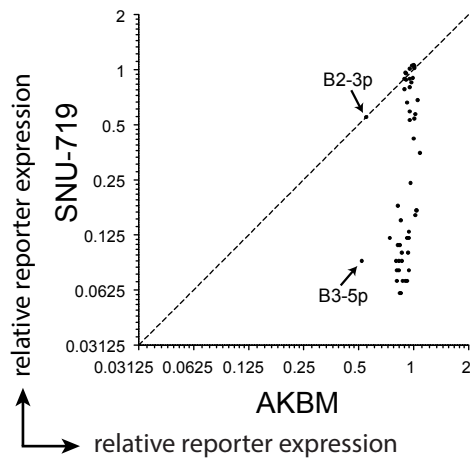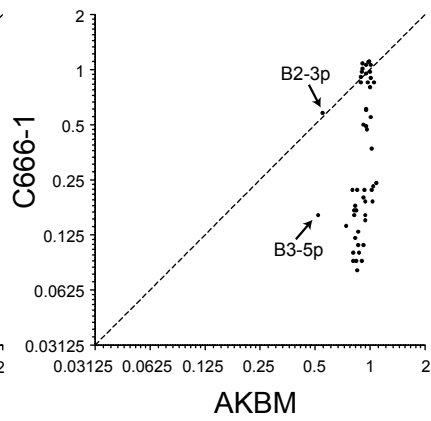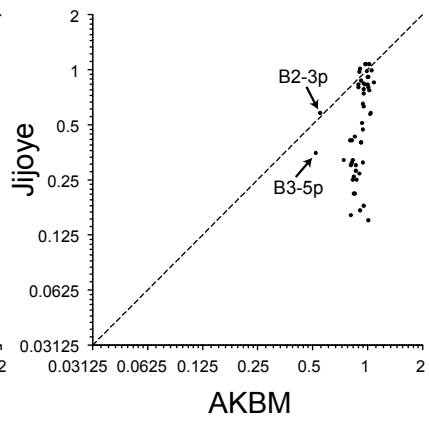

Supplement: Additional file 3: — Comparison of functional EBV miRNA expression between AKBM and other EBV+ cell lines. miRNA reporter expression (data also shown in Fig. 1) was compared between AKBM and the three other EBV+ cell lines. Every dot represents the relative reporter expression of a specific miRNA as a percentage of the control reporter. The dashed line indicates the diagonal (no difference in relative reporter expression between both cell lines) (PDF 91 kb) [file 12864_2016_2978_MOESM3_ESM.pdf]

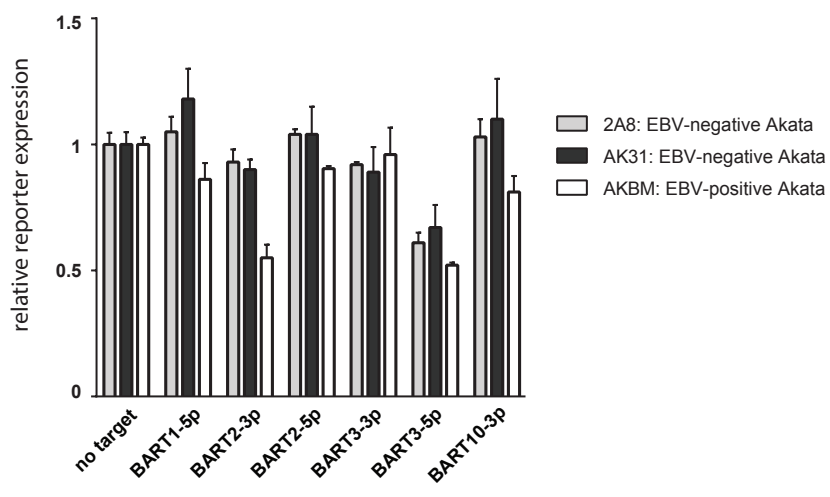

Supplement: Additional file 4: — Assessment of miRNA reporters in EBV− cells. Reporter activity for miR-BART2-3p, miR-BART3-5p and controls was compared between AKBM and the EBV− Burkitt’s lymphoma cell lines, 2A8 and AK31 (AKBM data also shown in Fig. 1). The miR-BART2-3p reporter is only downregulated in AKBM but not in the other two cell lines, suggesting that downregulation of the reporter is specific for EBV+ cells and thus likely due to miR-BART2-3p expression. The miR-BART3-5p reporter was downregulated in all three cell lines, suggesting the influence of a common host cell factor such as a human miRNA on reporter expression (PDF 101 kb) [file 12864_2016_2978_MOESM4_ESM.pdf]

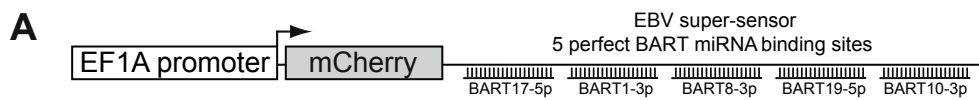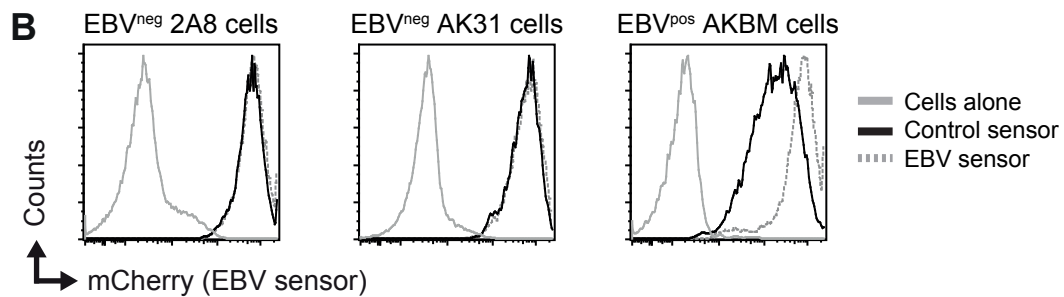

Supplement: Additional file 6: — AKBM cells are EBV positive. A) Schematic representation of the EBV super-sensor. Five perfect EBV miRNA target sites were cloned downstream of the mCherry reporter gene. The vector allows for sensing of EBV presence by monitoring the combined functional expression of 5 EBV miRNAs. B) EBV-positive AKBM cells and EBV negative AK31 and 2A8 cells were either left untreated (cells alone), were transduced with an mCherry-control vector (control sensor), or were transduced with the EBV sensor from a). Cells were monitored for mCherry reporter expression at 7 days post transduction by flow cytometry (PDF 165 kb) [file 12864_2016_2978_MOESM6_ESM.pdf]

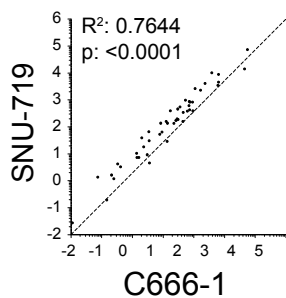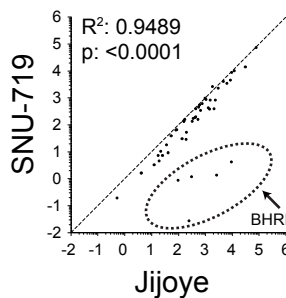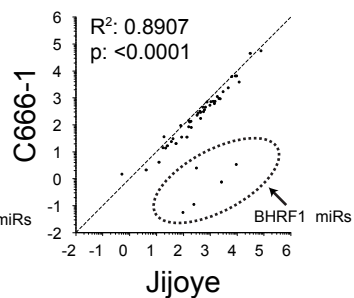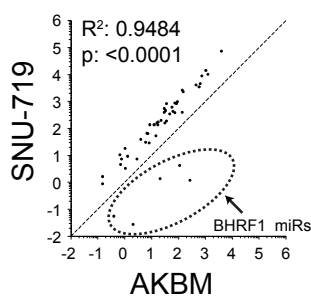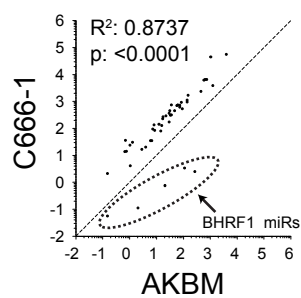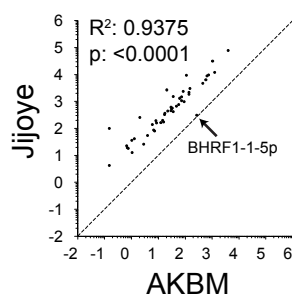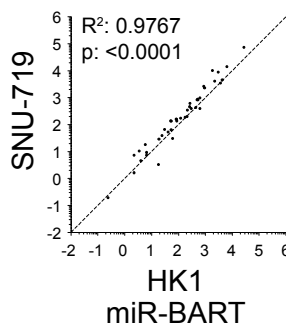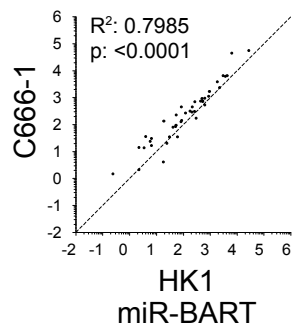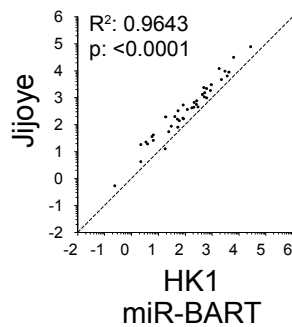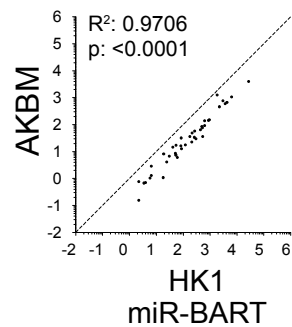

reads per million (10-log)

reads per million (10-log)

Supplement: Additional file 7: — Comparison of deep sequencing reads between different cell lines. Deep sequencing reads were compared between the four EBV+ cell lines and the EBV− cell line HK1-miR-BART. Every dot represents a single miRNA arm. BHRF1 miRNAs, BART2-3p and BART2-5p are not depicted in the HK1-miR-BART plots as these are not expressed in these cells. Pearson correlations R square and p-values are presented. Good correlations were observed between the read counts for BART miRNAs in all combinations of cell lines (PDF 157 kb) [file 12864_2016_2978_MOESM7_ESM.pdf]
